# Protocol for the isolation and characterization of porcine brain region-associated extracellular particles

**DOI:** 10.1371/journal.pone.0329985

**Published:** 2025-08-27

**Authors:** Abigail De Avila, Mayra Diaz, Bruno A. Cisterna, Solangel Castillo, Juan Pablo Lezana, Jay Molino, Luis Luis, Miryam Venegas-Anaya, Diego Reginensi

**Affiliations:** 1 Regenerative Therapies (↑), Faculty of Biosciences and Public Health, Universidad Especializada de las Américas (UDELAS), Panama City, Panama; 2 Regenerative Therapies (↓), Faculty of Medicine, Universidad de Panamá (UP), Panama City, Panama; 3 Doctorate Program in Biosciences and Biotechnology, Faculty of Sciences and Technology, Universidad Tecnológica de Panama (UTP), Panama City, Panama; 4 Department of Neuroscience and Regenerative Medicine, Medical College of Georgia, Augusta University, Augusta, Georgia, United States of America; 5 SAVAL Laboratory S.A, Santiago, Chile; 6 Molecular Observation and Research in Nanodynamics, Faculty of Biosciences and Public Health, Universidad Especializada de las Américas (UDELAS), Panama City, Panama; 7 Biomedical Engineering, Faculty of Engineering, Universidad Latina de Panama (ULATINA), Panama City, Panama; 8 Smithsonian Tropical Research Institute, Panama City, Panama; 9 Centro de Investigaciones Hidraulicas e Hidrotecnicas (CIHH) of Universidad Tecnologica de Panama, Panama City, Panama; 10 Center for Biodiversity and Drug Discovery, INDICASAT-AIP, City of Knowledge, Panama City, Panama; Instituto do Cancer do Estado de Sao Paulo / University of Sao Paulo, BRAZIL

## Abstract

Extracellular particles (EPs) are a heterogeneous pool of secreted messengers in cell-cell communication. The isolation of EPs from supernatants, biofluids, and solid tissues allows further research into understanding the role of EPs in physiological and pathological scenarios, which aids in the development of EP-based therapies in biomedicine. This article presents a straightforward, direct, and applicable method for isolating and characterizing EPs from three regions of the porcine brain: the cerebrum, cerebellum, and brainstem. The protocol is a method based on three steps: enzymatic treatment, differential centrifugation, and filtration/ultrafiltration to isolate the brain’s EPs. Analysis by scanning electron microscopy (SEM) and nanoparticle tracking analysis (ZetaView) revealed the enrichment of the brain’s EPs in the size range of 20–200 nm when isolated using this protocol. Additionally, CD63 and HSP70 expression was assessed by Western Blot without finding significant differences between the brain regions. This simple adapted method will help the understanding of extracellular ecosystems in the CNS and could have interesting implications in brain diagnosis and therapy.

## Introduction

Extracellular particles (EPs) is the preferred term for cell-derived multimolecular assemblies that range in size from nanometers to microns. This category includes both extracellular vesicles (EVs) and non-vesicular extracellular particles (NVEPs) [1–3].

There are several methods for isolating EPs, including sequential ultracentrifugation (UC) [4,5], gradient ultracentrifugation (DGUC) [4,5], asymmetric flow field-flow fractionation (AF4) [6,7], ultrafiltration (UF) [8,9], size exclusion chromatography (SEC) [10,11], immunoaffinity capture (IAC) [12], mass spectrometry [13], or microfluidic-based techniques [14]. In the filtration process, a suspension passes through a filter using methods such as gravity, centrifugation, or vacuum. Water and molecules smaller than the filter’s molecular weight cut-off pass through, while EPs larger than the cut-off are retained in the concentrated fluid compartment of the filter. This method effectively increases the particle number relative to the sample volume. It is also a low-cost, fast-processing alternative suitable for low-resource laboratories and developing countries.

It is essential to conduct studies like this, as they provide valuable insights into the presence of extracellular particles associated with EVs released near the site of action of the Cell-EV interaction within the tissue, as well as the intrinsic extracellular matrix composition. Small extracellular vesicles have been obtained from different solid tissue types, including tumoral tissue [15], lung tissue [16], kidney tissue [17], heart tissue [18], spleen tissue [19], liver tissue [20], adipose tissue [21], dental tissue [22] and brain tissue.

For brain tissue, the most commonly used methods for obtaining purified fractions of EVs are density gradient ultracentrifugation (DGUC) [23–38] and size exclusion chromatography (SEC) [36,39–41]. Certainly, the obtaining of subpopulations is an aspect to take into consideration for specific fractions analysis and a better understanding of each fraction’s functions, as it has been reported that the composition of small extracellular vesicles from the upper or lower limits of the ranges may differ due to surface area, volume, membrane components and soluble cargo (e.g., amount of proteins for EVs by 30 nm is 100 proteins, while EVs of 150 nm is 2,500 proteins) [42].

Different authors have obtained sEVs from (i) human brain tissue: sucrose DGUC is the technique applied by Vella et al. for frontal cortex [23] and You et al. for temporal lobe [43]; (ii) macaque whole brain EVs were obtained by DGUC [27]; (ii) mouse whole brain tissue: Brenna et al. [26] and Polanco et al. [38] used DGUC and (iii) rat forebrain by DGUC [28]. (v) Also, for human and mouse brain tissue (in the same studio): D’Acunzo et al. applied DGUC [32], and Matamoros et al. technique included DGUC for frontal cortex [29], and Muraoka et al. applied SEC [40]. Our goal is to characterize this heterogeneous population of EPs from solid brain tissue, and this report is the first study to obtain and report extracellular particles from porcine brain tissue.

In this protocol, we applied the “best practices recommendations” from MISEV 2023 for isolating extracellular vesicles from solid tissues. This includes guidelines on storage temperature and sample processing, the appropriate culture medium for harvesting extracellular particles (EPs), and the enzymatic dissociation of sliced tissue [44]. Here, we present an adapted protocol for isolating EPs within a size range of 20 nm to 200 nm, utilizing sample concentration techniques such as ultrafiltration. The samples were taken from three regions of the porcine brain: the cerebrum, cerebellum, and brainstem. The isolated EPs were characterized and quantified using scanning electron microscopy (SEM), nanoparticle tracking analysis, and immunoblotting techniques.

## Materials and methods

The protocol described in this article can be found on protocols.io (dx.doi.org/10.17504/protocols.io.j8nlk8y1wl5r/v1) and is included for printing as S5 File.

### Materials

#### Materials for brain EP isolation.

Collagenase type III (Cat# 215070401; MP Biomedicals), Hibernate-E Ca (Cat# M36101; Neuromics Inc), (Cat# 4906845001; Roche), cOmplete™ Protease Inhibitor Cocktail (Cat# 05892970001; Roche), phosphate-buffered saline (PBS; cat# P32200-10000.0; Research Products International), 10 kDa ultrafiltration tubes (Amicon; Cat # UFC901024; Millipore)

#### Materials for brain EPs characterization by electron microscopy and ZetaView analysis.

Formvar square mesh grids (Cat # 01753-F, Ted Pella), glutaraldehyde (Cat # G6257-100ML; Sigma Aldrich), uranyl acetate (UA) (Cat # 22400; Electron Microscopy Sciences).

#### Materials for brain EPs characterization by Western Blot analysis.

RIPA (N653-100ML, VWR), BCA assay kit (A65453, Pierce Thermo Scientific), 1.0 mm spacers (165311 Biorad), APS (1610700, Biorad), TEMED (1610801, Biorad), 25x protease inhibitors (11873580001, Roche), SDS (1610302, Biorad), Glycine (1610718, Biorad), Tris base (1610719, Biorad), Laemmli buffer (1610747, Biorad), 2-Mercaptoethanol (M3148-100ML, Sigma-Aldrich), Methanol (9070-68, JT Baker), gel releasers (1653320, Biorad), PVDF membrane (Immobilon-P PVDF membrane, IPVH00010), blot roller (1651279, Biorad), Ponceau S (ab146313, Abcam), Tween-20 (P1379-100ML, Sigma Aldrich), Tris HCl (IB70162, IBI Scientific), NaCl (S5886-500G, Sigma Aldrich), HCl (Cat # 1003172500; Millipore), Non-Fat Dry Milk (NFDM) (1706404, Biorad), Anti-CD63 (Cat # ab134045; Abcam), Anti-HSP70 (Cat # ab181606; Abcam), Anti-NeuN (Cat # MAB377; Sigma-Aldrich), secondary antibody Anti-rabbit (ab6721; Abcam), chemiluminescent substrate (926–95010, LICOR).

### Methods

#### Isolating specific regions of the porcine brain.

The porcine brain tissues were extracted from 6 to 8-month-old porcine weighing approximately 118 kg immediately after being sacrificed at Marcello, S.A. (Panama City, Panama). The brains were quickly frozen on dry ice and transferred to −80 ºC for preservation, minimizing freeze-thaw degradation. After two days, the brains were thawed to remove blood vessels and were dissected to obtain three specific brain regions: the cerebrum, cerebellum, and brainstem. Each dissected area was cut into small pieces, approximately 1 cm³, for subsequent procedures. Brain pieces were obtained for EP isolation and others were pulverized in a dry mortar with a pestle to obtain homogenized tissue [45,46]. The use of post-mortem porcine brain tissue was exempted from animal-use approval by the Animal Research and Welfare Ethics Committee of the University of Panama.

#### Isolation of EP from brain-region tissue.

**Fig 1:**
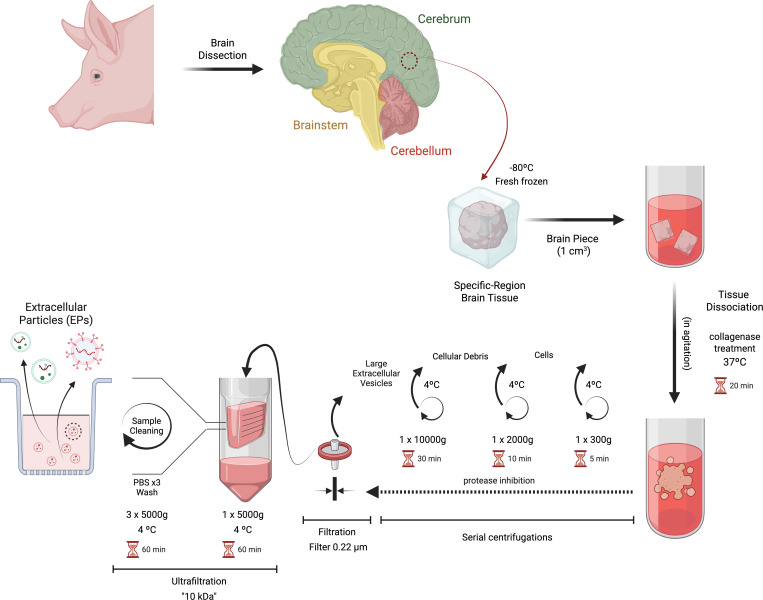
Schematic of isolating extracellular particles (EPs). EPs were isolated from porcine brain tissue through the following steps: (i) enzymatic treatment using collagenase type III for tissular dissociation, (ii) protease and phosphatase inhibition for ensuring protein integrity, (iii) differential centrifugation to remove tissue, cellular debris and large EPs, and (iv) filtration to enrich the sample in EPs smaller than 200 nm, (v) followed by ultrafiltration with a 10 kDa molecular weight cut-off to concentrate the sample and obtain extracellular particles from different brain regions (Created with Biorender.com, accessed Dec. 2024).

Brain pieces of porcine brain were randomly selected from three brain regions: the cerebrum, cerebellum, and brainstem. These pieces were sliced and treated with 75 U/ml of collagenase type III (Cat# 215070401; MP Biomedicals), in Hibernate-E Ca (Cat#M36101; Neuromics Inc), using a ratio of 800 μl of the solution for every 100 mg of brain tissue. The mixture was incubated at 37°C while shaking at 55 rpm for 20 min. Following this treatment, brain cells were removed by centrifuging the mixture at 300 x g for 5 min at 4°C. The supernatant was then transferred to a polycarbonate tube containing PhosSTOP™ (Cat# 4906845001; Roche) and cOmplete™ Protease Inhibitor Cocktail (Cat# 05892970001; Roche) in phosphate-buffered saline (PBS; cat# P32200-10000.0; Research Products International). This was centrifuged at 2,000 x g for 10 min at 4°C to eliminate cellular debris. Next, to remove large debris and vesicles >200 nm, enriching for EPs within the 20–200 nm range, the supernatant underwent a second centrifugation at 10,000 x g for 30 min at 4°C and then clarified through a 0.22 μm filter. The clarified supernatant was placed in 10 kDa ultrafiltration tubes (Amicon; Cat # UFC901024; Millipore) and centrifuged at 5,000 x g for 1 h to concentrate the extracellular particles, with samples being mixed every 15 min during this process. Finally, the concentrated supernatant was washed three times by adding 15 ml of PBS and centrifuging at 5,000 x g for 1 h at 4°C to obtain the brain EPs [23,47]. The schematic representation of the isolation of EP from brain-region tissue is shown in Fig 1.

#### Characterization of brain EPs by scanning electron microscopy (SEM).

We mounted the brain EPs onto formvar square mesh grids (Cat # FF300-CU; Electron Microscopy Sciences). Three different fixation protocols were evaluated: Keerthikumar’s Protocol [48] (Protocol A), Polanco’s Protocol [38] (Protocol B), and Lunavat’s Protocol [49] (Protocol C). Samples were charged onto the dark and shiny side of the grids, and the excess of liquid was paper blotted away between each step. Prior SEM analysis using the Quattro Environmental Scanning Electron Microscope (Thermo Fisher Scientific), all prepared grids were kept away from light sources.

Protocol A: 10 μL of EPs sample was placed over TEM grids for five minutes. Excess residue was blotted away. 10 μL of 2% uranyl acetate (UA) (Cat # 22400; Electron Microscopy Sciences) were pipetted twice over the grid for negative staining for 1 minute [48]. Following Keerthikumar’s protocol, a total of 436 EPs were acquired from 38 micrographs during the electron microscopy procedure. This total was distributed as follows: 90 EPs from the cerebrum, 149 EPs from the cerebellum, and 197 EPs from the brainstem.
Protocol B: Samples from each region were fixed using 2% glutaraldehyde (Cat # G6257-100ML; Sigma Aldrich) for 30 min. A 1:1 ratio of sample to fixation solution was prepared, and 10 µL of the fixed EP was added to the TEM grids, where it was allowed to absorb for five minutes. Each grid was then washed twice with a droplet of sterile water. After washing, 10 µL of 1.5% UA was applied for 1 min, ensuring it was protected from light. [38]. A total of 362 EPs distributed across 39 micrographs were acquired on the grids prepared according to Polanco’s protocol. The distribution of EPs was as follows: 108 for the cerebrum, 128 for the cerebellum, and 126 for the brainstem.
Protocol C: The grids were exposed to ultraviolet radiation for 15 minutes on their dark-shiny side. EPs samples in a volume of 15 µL were incorporated into the grids for 10 minutes. Samples were fixed by adding 15 µL of 2.5% glutaraldehyde for 10 min. Fixed samples were stained with 15 µL of 2% UA for 10 minutes [22]. In total, 396 EPs were identified from 47 micrographs, distributed as follows: 137 EPs for the cerebrum sample, 138 EPs for the cerebellum sample, and 121 EPs for the brainstem.

Images were taken in Quattro scanning electron microscope (SEM), in transmission mode, for all the samples prepared, operating at 30.00 kV. The overall magnification was 50,000 x – 100,000 x, with a current of 0.7 nA and working distance (WD) 9.3 mm to 10.9 mm. To enhance visualization, Dark-Field 4th ring and HAADF (High-Angle Annular Dark-Field) modes were selected. The electron microscopy was conducted at the Instituto de Investigaciones Científicas y Servicios de Alta Tecnología (INDICASAT-AIP) in Panama.

Ultrastructure measurements included the estimation of 334, 415, and 444 EPs from cerebrum, cerebellum, and brainstem, respectively. The diameter of each EP was calculated as the median of a pair of diameters for both round and elliptical shapes. In this analysis, only samples that met specific selection criteria were included: defined borders, non-clustering, and opacity due to the contrast provided by negative staining. Quantification and measurement assessments were performed manually using ImageJ version 1.54f.

#### Characterization of brain EPs by nanoparticle tracking analysis (NTA).

For NTA, the ZetaView (Particle Metrix, North Carolina, USA) instrumentation was validated using PS100 standard beads prior to the analysis of samples. Then, the EPs samples were gently vortexed and centrifuged at 10,000 rpm for 30 s. Each sample was diluted in particle-free water (MilliQ) for a total of 100–300 particles per field (Cerebrum EPs dilution factor: 50,000; Cerebellum EPs and Brainstem EPs dilution factor: 100,000). For each measurement, consider size distribution mode, 1 cycle and 11 positions with the following settings: (i) video resolution: High, (ii) laser wavelength: 488 nm, (iii) filter wavelength: scatter, (iv) camera sensitivity for all samples: 82.0, (v) shutter: 95, and (vi) cell temperature: 24°C. After capture, analyze the videos using the build-in ZetaView Software 8.06.01 with specific analysis parameters: maximum particle area: 1000, minimum particle area: 6, and minimum particle brightness: 20 [50].

#### Characterization of EPs by Western Blot.

For brain tissue, a small ~20 mg piece was placed in PBS with inhibitors of phosphatase and protease, homogenized with a Wheaton tissue grinder and placed at −80°C for later use. Samples were lysed by radioimmunoprecipitation assay with RIPA lysis buffer (Cat # N653-100ML; VWR) supplemented with protease (Cat# 11873580001; Roche) and phosphatase (Cat # 4906845001; Roche) inhibitors at 4°C. Samples were sonicated for 2 min at room temperature, and lysis was completed by incubation at 4°C overnight. Cellular debris and large EPs were then removed by centrifugation at 12,000 rpm at 4°C for 5 min.

Using the BCA assay kit (Pierce; Cat # A65453; Thermo Scientific), the overall protein concentration of the samples was determined. Prepare a diluted stock of the protein supernatant (e.g., 1:10 dilution) for analysis. Analyze the samples in duplicates. Protein concentrations may vary between samples (e.g., 2.04–4.12 µg/µl for native brain cerebrum); therefore, it is essential to perform the assay for each experiment. 20 µg of proteins were loaded onto each gel lane. For the identification of tetraspanin proteins (e.g., CD63), the samples were prepared for loading using Laemmli buffer (4x; Cat # 1610747; Biorad) without a reducing agent. Laemmli buffer was diluted 1:1 with dH_2_O to prepare a 2x working stock. Prior to sample loading in the gel, protein samples were mixed with equal parts of the 2x Laemmli buffer to achieve a final 1x buffer concentration, and samples were heated in a dry bath incubator for 5 min at 95°C. For the identification of other proteins (e.g., HSP70, NeuN), the samples were additionally prepared with Laemmli buffer, including 355 mM 2-Mercaptoethanol (Cat # M3148-100ML; Sigma-Aldrich).

For electrophoresis, samples were loaded onto 10% polyacrylamide gels (10% TGX Stain-Free Fast Cast; Cat # 1610183; Biorad). The parameters were set at 100 V for 2 h. Proteins were transferred from the gel to a PVDF membrane (Immobilon-P PVDF membrane; Cat # IPVH00010; Millipore) with the following parameters: for two mini-gels, the transfer system (Transblot Turbo, Biorad) was set in one cassette at 25V, 1.0A for 60 min. The gel was washed in dH_2_O, and the total protein retained in the gel was visualized using Coomassie Stain (Cat # B43000-25.0; RPI). After transfer, the total protein was visualized using Red Ponceau stain (Cat # ab146313; Abcam).

The membrane was blocked with 5% fat-free milk (NFDM; Cat # 1706404; Biorad) in Tris-buffered saline with Tween 20 (TBS-t) buffer for 1 h at 100 rpm at room temperature. Following this, the membrane was incubated with primary antibodies, which were diluted in 5% NFDM, for 16 h at 100 rpm at 4°C. After incubation, the membrane was washed three times with TBS-t. The primary antibodies used were: Anti-CD63 (1:1,000; Cat # ab134045; Abcam), Anti-HSP70 (1:1,000; Cat # ab181606; Abcam), and Anti-NeuN (1:1,000; Cat # MAB377; Sigma-Aldrich). Next, the membrane was incubated with a secondary antibody (anti-rabbit, 1:1,000; ab6721; Abcam) for 1 h at 100 rpm at room temperature and after incubation, the membrane was washed three times with TBS-t. Afterward, the membrane was developed with a chemiluminescent substrate (Cat # 926–95010; LICOR) for 5 minutes at room temperature without shaking. Visualization was performed using a blot scanner (C-DiGit; Licor) in high resolution after a 12-minute exposure.

### Statistical analysis

Data are presented as mean ± Standard Error of the Mean (SEM) of at least three independent experiments (unless indicated). Data was analyzed using one-way ANOVA with Tukey-Kramer post hoc analysis (p < 0.05). All analysis and graphing of results were performed using GraphPad Prism 10 software.

## Results

### Isolation of EPs from different regions of the brain

**Fig 2:**
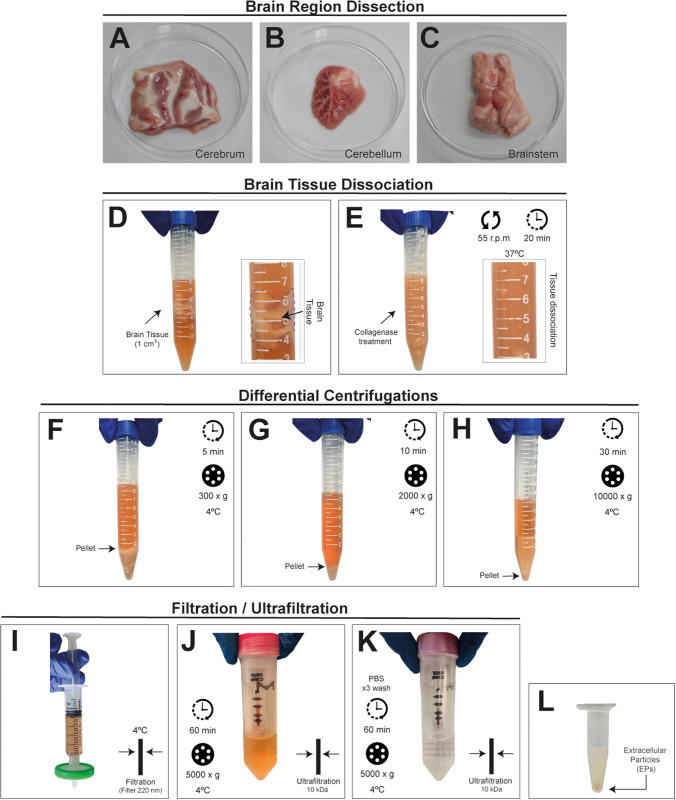
Stepwise schematic for isolation of extracellular particles from porcine brain tissue. Tissue composition and structural features of dissected brain regions: (A) the cerebrum displays organized gray and white matter with prominent sulci and gyri; (B) the cerebellum shows characteristic folia and underlying white matter; (C) the brainstem is composed predominantly of white matter tracts with scattered gray matter nuclei; (D) The sliced tissue is placed in the medium containing collagenase type III; (E) The preparation was then incubated at 37 °C to initiate the dissociation of the tissue, following collagenase treatment, the tissue undergoes differential centrifugation of the supernatant: (F) at 300 x g, (G) at 2000 x g, (H) at 10000 x g. (I) The filtration process employs a filter with a pore size of 220 nm, and (J) ultrafiltration (10 kDa) is used to concentrate the samples based on molecular weight. (K) The samples are then washed three times with filtered PBS to ensure removal of residual medium, utilizing an ultrafiltration system. (L) Finally, after the ultrafiltration process, we obtain brain extracellular particles (EPs).

After extracting the porcine brain, each region was carefully dissected. The cerebrum, the largest part of the brain, is characterized by its extensive surface area and convolutions. In contrast, the cerebellum is the smallest tissue and has a distinct branched structure. The brainstem, which is the densest tissue, was separated from the cerebrum by making an incision along the corpus callosum. This region includes the midbrain, pons, and medulla oblongata, excluding the initial section of the medulla (Fig 2A–C). The protocol consisted of the following steps: (i) dissection of brain regions, (ii) enzymatic disaggregation of the tissue by incubation in collagenase III with calcium-free medium, (iii) removal of cell debris through differential centrifugation, (iv) elimination of large extracellular particles via 200 nm filtration and (v) enrichment of small EPs using 10kDa ultrafiltration. The tissue samples were processed based on a predefined weight range to ensure uniformity among the different brain regions. During digestion with collagenase type III, we observed a high density and intense pigmentation in the samples (Figs 2D and E). Through differential centrifugation a supernatant containing the small EPs was obtained (Fig 2F and 2H); to concentrate and enrich the small EPs, we used a syringe filtration method (Fig 2I). After concentration using a membrane with a 10 kDa molecular weight cutoff (Fig 2J), the media was exchanged for sterile-filtered PBS to enhance sample cleanliness prior to characterization (Fig 2K). The final sample consists of a pool of extracellular particles (EPs) from the cerebrum, cerebellum, and brainstem (Fig 2L). This experimental part is the adaptation of different protocols for isolating extracellular particles [31,47,51].

#### Morphological characterization of EPs from different regions of the brain.

**Fig 3:**
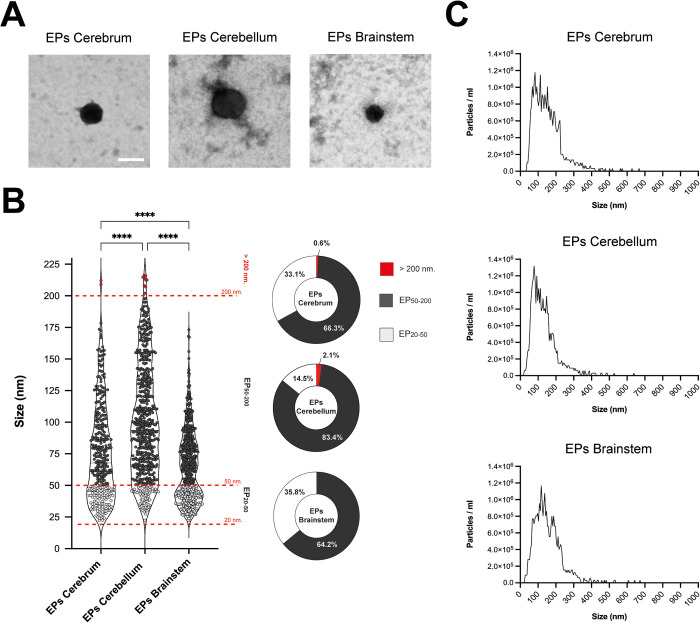
Morphological characterization and quantification of brain EPs. (A) **Scanning electron microscopy of brain EPs.** The images show extracellular particles obtained from the three brain regions: cerebrum, cerebellum, and brainstem; (B) **Dot plot representation of EP size from region-specific brain (ImageJ counting).** Brain EP’s size in two ranges were observed: EP_50-200_ (range: 50 to 200 nm) and EP_20-50_ (20 to 50 nm). Data is represented protocol-independent as the total EPs per region: cerebrum, cerebellum, and brainstem; red bars denote the range cut per EPs diameter size; (C) **Nanoparticle Tracking Analysis by ZetaView.** The graphs display the overall size distribution of particles per milliliter for each brain region. Significant differences were determined between all groups shown by one-way ANOVA with Tukey-Kramer post hoc analysis (p < 0.05), with ****p < 0.0001. In (A): Scale bar = 100 nm.

The extracellular particles (EPs) were negatively stained onto formvar grids for morphological and size characterization by Scanning Transmission Electron Microscopy (STEM). EPs primarily exhibited a round shape, although a minor percentage displayed elliptical shapes. Scanning Transmission Electron Microscopy (STEM) is a hybrid method of TEM and SEM that lacks resolution in the identification of smaller structures. The images obtained did not exhibit the typical morphology seen in TEM images because STEM (SEM in transmission mode) provides shape contrast but at a lower resolution than conventional TEM. No significant inter-method (protocol A, B, and C) difference was found in the EPs roundness morphology, size range, and background uniformity (Fig 3A).

The individual values plot of the samples from protocols A, B, and C, denoted the presence of EPs of different diameters. The details are explained below:

PROTOCOL A: The size range of EPs was 29.8 to 209 nm (mean range: 179.2 nm) in the cerebrum, 30.2 to 216.2 nm (mean range: 185.9 nm) in the cerebellum, and 22.5 to 121.4 nm (mean range: 98.9 nm) in the brainstem, with average sizes of 93.08 nm ± 43.54, 106.86 nm ± 44.93, and 56.49 nm ± 22.05, respectively.
PROTOCOL B: The size range was 24.8 to 212.1 nm (mean range: 187.3 nm) in the cerebrum, 32–202 nm (mean range: 170 nm) in the cerebellum, and 23.9 to 173.2 nm (mean range: 149.3 nm) in the brainstem, with average sizes of 79.78 nm ± 34.17, 90.13 nm ± 43.98, and 75.42 nm ± 33.03, respectively.
PROTOCOL C: The size range was 19.6 to 157.2 nm (mean range: 137.6 nm) in the cerebrum, 22.4 to 215.8 nm (mean range: 193.4 nm) in the cerebellum, and 27.3 to 167.3 nm (mean range: 140.1 nm) in the brainstem, with average sizes of 60.39 nm ± 30.55, 100.37 nm ± 48.01, and 78.21 nm ± 27.33, respectively.

Overall, we observed no significant differences in the results obtained between the three protocols: protocol A, protocol B, and protocol C (S1A Fig). The raw data associated is included in the S1B File.

The EPs size was measured and analyzed by using ImageJ. Size range was determined and EPs were grouped into EP_20–50_ and EP_50–200_ populations, obtaining the following results: for cerebrum EPs (n = 335), the size range was 19.6 to 212.1 nm, comprising 33.1% for the EP_20–50_ range (20–50 nm) and 66.3% for the EP_50–200_ range (50–200 nm); for cerebellum EPs (n = 415), the size range was 22.4 to 216.2 nm, including 14.5% for EP_20–50_ and 83.4% for EP_50–200_; and for brainstem EPs (n = 444), the size range was 22.5 to 173.2 nm, accounting for 35.8% for EP_20–50_ and 64.2% for EP_50–200_. This analysis indicates the highest concentration of EPs in the EP_50–200_ range across all the brain tissue regions (Fig 3B). More details can be found in the supporting information S2 File.

The size distribution data of brain EPs were obtained by Nanoparticle Tracking Analysis with Particle Metrix ZetaView and the results were presented as a diameter (nm) versus measured particles graph (Fig 3C). The most abundant particle quantity per size (mode) were observed at 82.5 nm (1.18 x 10^6^ particles/ml), 77.5 nm (1.32 x 10^6^ particles/ml), and 117.5 nm (1.17 x 10^6^ particles/ml) for cerebrum EPs, cerebellum EPs and brainstem EPs, respectively. In our results, extracellular particles over the concentration of 2 × 10^5^ particles/ml were distributed in the following size ranges: (i) Cerebrum EPs: 47.5–222.5 nm, representing 86.71% of particles from cerebrum, (ii) Cerebellum EPs: 37.5–207.5 nm, representing 88.11% of EPs from cerebellum, and (iii) Brainstem EPs 47.5–237.5 nm, representing 89.01% of EPs from brainstem; NTA showed a drop-off in counts above ~200 nm. More information associated with the NTA data can be found in the supporting information S3 File.

#### Biochemical characterization of EPs from different regions of the brain.

**Fig 4:**
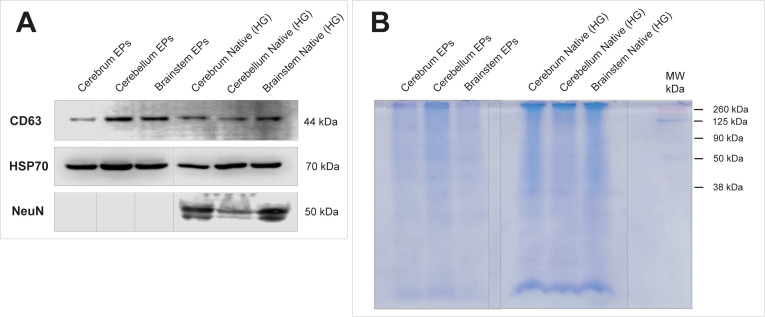
Western Blot analysis of three specific regions of the brain: cerebrum, cerebellum, and brainstem. (A). CD63, HSP70 and NeuN were analyzed by immunoblotting. We observed the expression of CD63 and HSP70 in the brain’s EP, while NeuN (negative control) was not expressed in brain’s EPs. Irrelevant lanes have been removed and main fragments kept aligned. (B) The total protein levels in the three regions for brain homogenate and associated brain’s EPs were visualized using Coomassie Brilliant blue in the polyacrylamide gel.

Western blot analysis of EPs extracted from the cerebrum, cerebellum, and brainstem revealed the presence of CD63 and HSP70 markers. These proteins are commonly found in the composition of EVs in general [23,25,27,52]. The known markers for extracellular vesicles studied correspond to the main classifications for biochemical characterization of EPs as recommended by MISEV23: (i) multi-pass transmembrane proteins, which includes the tetraspanin CD63, this type of protein is associated with the plasma membrane, and (ii) cytosolic proteins in EPs, including the Heat Shock Protein of 70kDa (HSP70) [44]. The anti-NeuN, which targets a neuronal nuclear antigen, was used in an experiment designed to (i) compare native tissue against extracellular particle (EP) preparations from each brain region of study, and (ii) confirm the absence of neuronal nuclei in the EP fractions. NeuN immunoreactivity was detected in native tissue samples from cerebrum, cerebellum, and brainstem, confirming the presence of cellular components. In contrast, EP preparations did not exhibit NeuN labeling, indicating the absence of neuronal nuclei and supporting the effective removal of cellular debris during sample processing (Fig 4A). The total proteins of extracellular particles from the cerebrum, cerebellum, and brainstem, as well as from native brain tissue, were visualized using Coomassie Brilliant Blue staining. This revealed clear differences in protein banding patterns between native brain tissue and extracellular particles. (i) Distinct bands were observed with some present exclusively in the native tissue and others uniquely found in the extracellular particle sample; (ii) while minor variations were noted, overall protein profiles remained consistent across the three brain regions (Fig 4B).

The raw blots associated can be found in S4 Raw Images.

## Discussion and conclusions

Isolating extracellular particles (EPs) from biological tissues remains a significant challenge, prompting ongoing efforts to refine isolation techniques. Porcine brains, commonly regarded as biological waste in slaughterhouses, represent a potentially valuable resource for biomedical research. In this study, we propose a novel application of porcine brain tissue in the biomedical field. This approach is supported by previous studies that have retrieved brain tissue from post-mortem pigs and used it for various neuroscience-related research [46,53]. This study presents a protocol to obtain EPs from three distinct brain regions: the cerebrum, cerebellum, and brainstem. The methodology incorporates enzymatic digestion, sequential centrifugation, and a combination of filtration and ultrafiltration.

This is the first study to isolate extracellular particles from porcine brain tissue. It has been reported that EVs can be obtained from porcine biofluids, cell culture and tissue: cerebrospinal fluid [54,55], blood plasma [54], milk [56], seminal fluid [54,57], uterine luminal fluid [58], skeletal muscle-derived cells [59,60] and adipose tissue-derived MSC [61]. Ultrafiltration (UF) is a widely used method prior to sample fractioning by size exclusion chromatography or density gradient ultracentrifugation [9]. It has been reported that UF itself can be useful as an alternative to UC because it leads to higher proteins, lipids, cytokines, and small EVs yield for industrial applications [62]. In our study, ultrafiltration was preceded by sequential centrifugation and filtration for debris removal, considering that our samples come from tissue and not biofluid or culture supernatant. The isolation of small EVs from brain tissue of other species has been well established and requires further steps like DGUC [23,25–30,34,35,43,63], SEC [36,39,40], affinity capture [63] and precipitation [24]; in our case, to study brains EPs, including small EVs and NVEPs.

The resulting brain EPs measure between 20 and 200 nm in size. There were similarities in the size distribution observed between electron microscopy (EM) and nanoparticle tracking analysis (NTA). In our results, the NTA showed a drop-off in counts above ~200 nm; while NTA (by both technologies, NanoSight and ZetaView) can detect particles across a broad range, it has been reported to underrepresent or fail to detect extracellular vesicles with peak diameters below 60 nm [50]; in contrast to EM, which has a minimal detection limit of particles by 20 nm [42].

Brain EPs exhibit the presence of CD63 and HSP70 markers, with no substantial variation observed across the different brain regions. In our case, we observed the expression of CD63 in brain EP, which is also expressed in the EV isolation in other studies from solid brain tissue [25,52,63]. HSP70 immunoreactivity has been confirmed in extracellular vesicles from brain tissues, as in other reports [28,64].

Further research is required to characterize EPs within the central nervous system (CNS) and to differentiate among extracellular vesicle (EV) subtypes—such as small EVs and small ectosomes—as well as non-vesicular extracellular particles (NVEPs) like exomeres and supermeres. The challenge of isolating brain EPs extends beyond research, holding potential implications for clinical applications. This streamlined protocol may facilitate the development of cost-effective strategies for EP isolation.

## Supporting information

S1A Fig Scanning electron microscopy (SEM), transmission mode, of region-brain EPs. EPs preparations of three different protocols: Protocol A, Keerthikumar et al.; Protocol B, Polanco et al.; and Protocol C, Lunavat et al. for ultrastructure analysis (SEM). The representative images of the morphological analysis, all the isolated samples from the porcine brain displayed the presence of brain EPs (white arrows), presented a rounded shape, continuous edges and a defined negative staining; while, particles that presented irregular shape, fragmented structure or diffuse edges are not considered, like brain EPs (red arrows). The individual values plot of the samples for protocol/region brain area showing the size distribution of EPs isolated from brain region-specific tissue for each protocol. Significant differences were determined between all groups shown by one-way ANOVA with Tukey-Kramer post hoc analysis (p < 0.05). Scale bar = 100 nm.
(TIF)

S1B File Raw data associated to Scanning Electron Microscopy results for each staining protocol: Protocol A (Keerthikumar’s), Protocol B (Polanco’s), and Protocol C (Lunavat’s). (XLSX)

S2 File Raw data of Scanning Electron Microscopy from Cerebrum EPs, Cerebellum EPs, and Brainstem EPs. (XLSX)

S3 File Raw data of Nanoparticle Tracking Analysis, ZetaView Reports. (XLSX)

S4 Raw Images Raw western blot membranes and gel. (TIF)

S5 File Stepwise protocol through protocols.io. (PDF)
