# Supplementary figures and images for: Protocol for the isolation and characterization of porcine brain region-associated extracellular particles
Source: PLoS One. 2025 Aug 27;20(8):e0329985. doi: 10.1371/journal.pone.0329985 (PMC12385349; doi:10.1371/journal.pone.0329985)

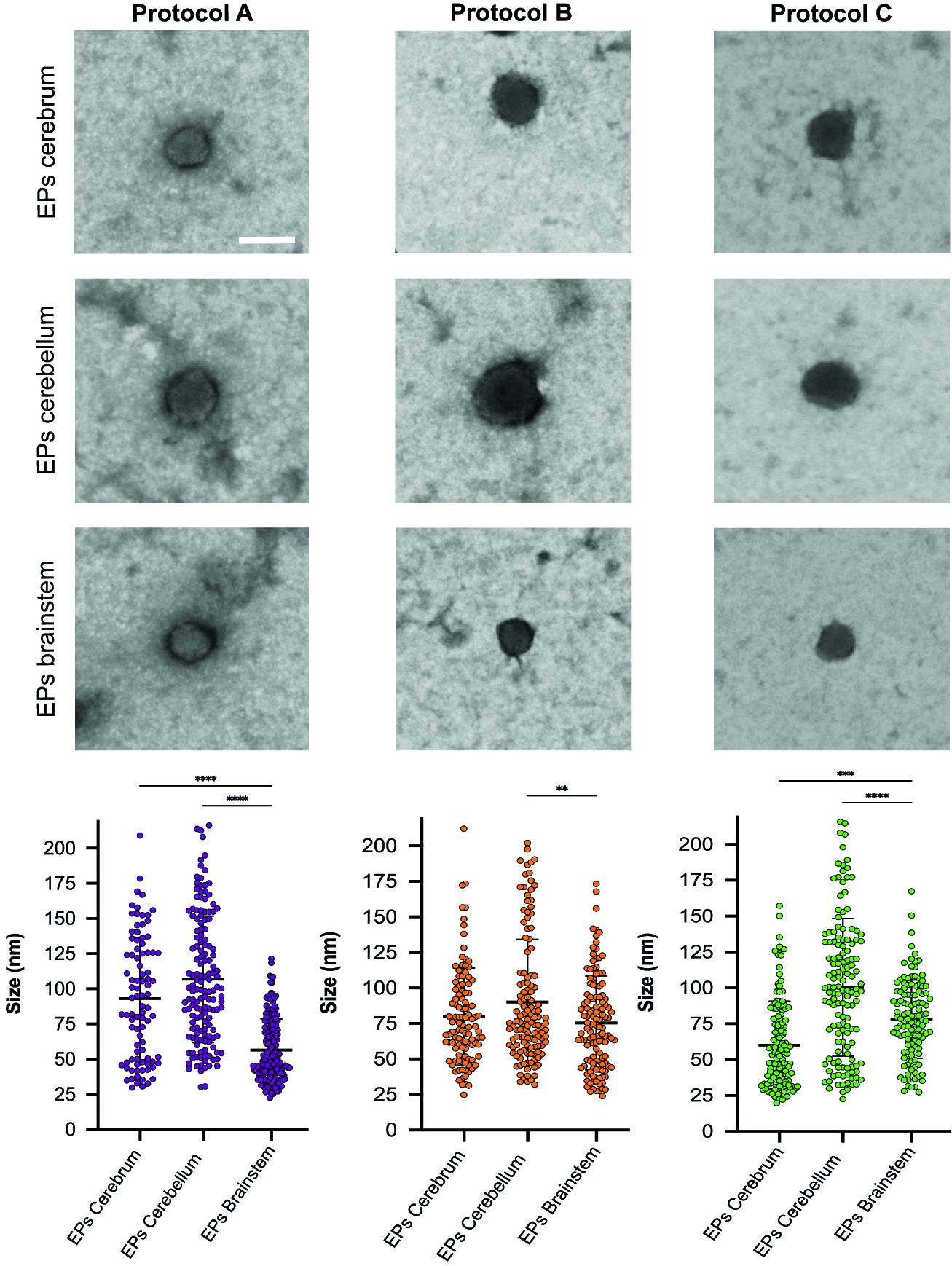

Supplement: S1A Fig — EPs preparations of three different protocols: Protocol A, Keerthikumar et al.; Protocol B, Polanco et al.; and Protocol C, Lunavat et al. for ultrastructure analysis (SEM). The representative images of the morphological analysis, all the isolated samples from the porcine brain displayed the presence of brain EPs (white arrows), presented a rounded shape, continuous edges and a defined negative staining; while, particles that presented irregular shape, fragmented structure or diffuse edges are not considered, like brain EPs (red arrows). The individual values plot of the samples for protocol/region brain area showing the size distribution of EPs isolated from brain region-specific tissue for each protocol. Significant differences were determined between all groups shown by one-way ANOVA with Tukey-Kramer post hoc analysis (p < 0.05). Scale bar = 100 nm. (TIF) [file pone.0329985.s001.tif]

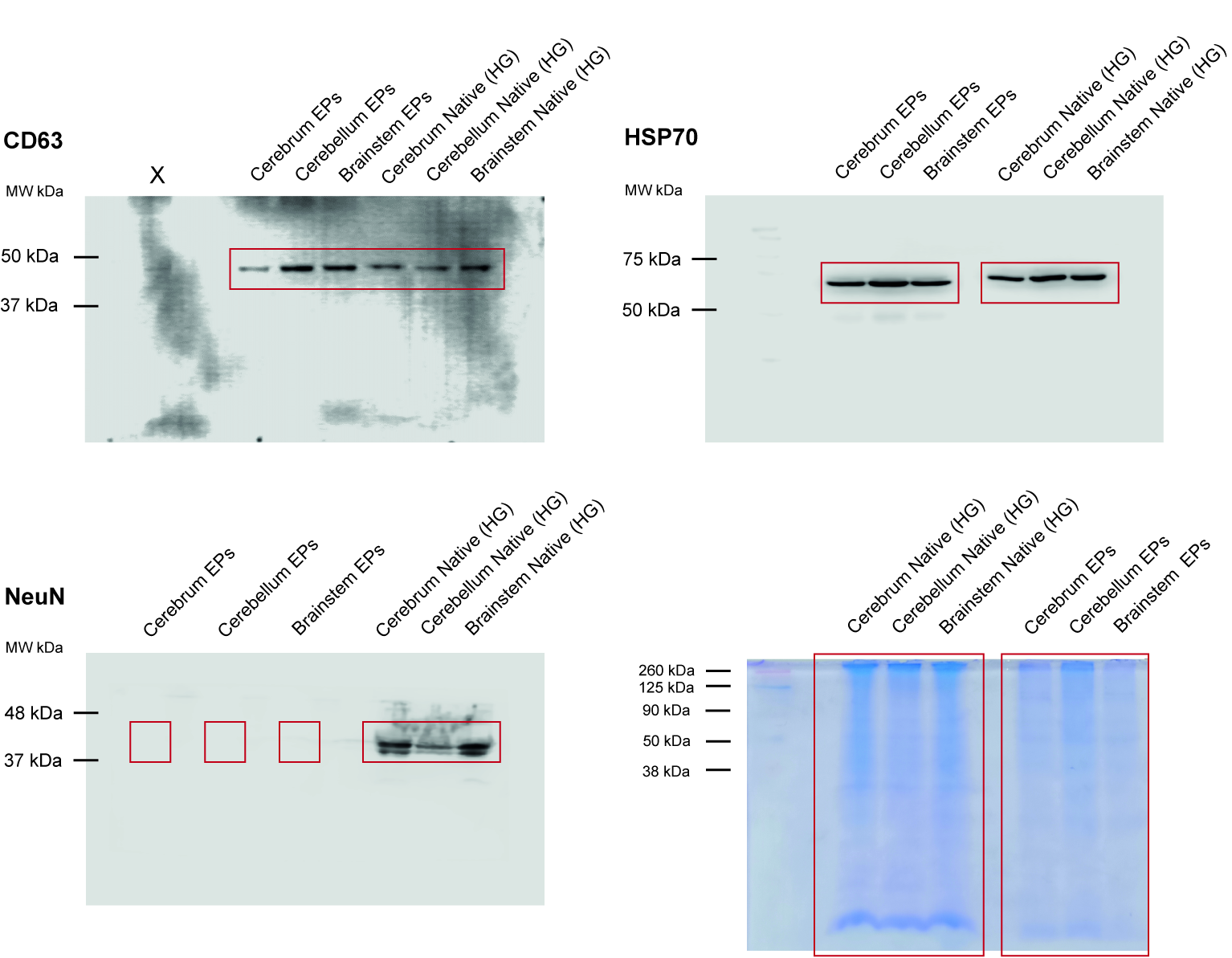

Supplement: S4 Raw Images — (TIF) [file pone.0329985.s005.tif]
