# Supplementary material for: Protocol for the isolation and characterization of porcine brain region-associated extracellular particles
Source: PLoS One. 2025 Aug 27;20(8):e0329985. doi: 10.1371/journal.pone.0329985 (PMC12385349; doi:10.1371/journal.pone.0329985)
Supplement: S5 File — (PDF) [file pone.0329985.s006.pdf]

# Protocol for the Isolation and Characterization of Porcine Brain Region-Associated Extracellular Particles.

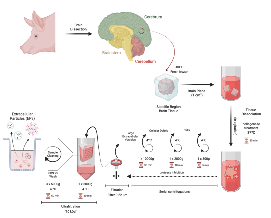

RESERVED DOI:
10.17504/protocols.io.j8nlk8y1wl5r/v1

Abigail De Avila *[1,2,3], Mayra Díaz *[1], Bruno A. Cisterna[4], Solangel Castillo[1,2], Juan Pablo Lezana[5], Jay Molino[6], Luis Luis[1,7], Miryam Venegas-Anaya ¶[3,8,9], Diego Reginensi ¶[1,2,7,10]

[1]Regenerative Therapies (↑), Faculty of Biosciences and Public Health, Universidad Especializada de las Américas (UDELAS), Panama.;
[2]Regenerative Therapies (↓), Faculty of Medicine, Universidad de Panamá (UP), Panama, Panama.;
[3]Doctorate Program in Biosciences and Biotechnology, Faculty of Sciences and Technology, Universidad Tecnológica de Panama (UTP);
[4]Department of Neuroscience and Regenerative Medicine, Medical College of Georgia, Augusta University, Augusta, GA, USA.;
[5]SAVAL Laboratory S.A, Santiago, Chile.;
[6]Molecular Observation and Research in Nanodynamics, Faculty of Biosciences and Public Health, Universidad Especializada de las Américas (UDELAS), Panama, Panama.;
[7]Biomedical Engineering, Faculty of Engineering, Universidad Latina de Panama (ULATINA), Panama, Panama.;
[8]Smithsonian Tropical Research Institute, Roosevelt Ave. Tupper Bldg. 401, Panama, Panama;
[9]Centro de Investigaciones Hidraulicas e Hidrotecnicas (CIHH) of Universidad Tecnologica de Panama.;
[10]Center for Biodiversity and Drug Discovery, INDICASAT-AIP, City of Knowledge, Panama, Panama.

Abigail De Avila *: Both authors contributed equally.;
Mayra Díaz *: Both authors contributed equally.;
Miryam Venegas-Anaya ¶: Corresponding Author;
Diego Reginensi ¶: Corresponding Author;

Diego Reginensi
Univ. de Panamá (UP), Univ. Especializada de Las Americas

**Protocol Info:** Abigail De Avila *, Mayra Díaz *, Bruno A. Cisterna, Solangel Castillo, Juan Pablo Lezana, Jay Molino, Luis Luis, Miryam Venegas-Anaya ¶, Diego Reginensi ¶ . Protocol for the Isolation and Characterization of Porcine Brain Region-Associated Extracellular Particles.. **protocols.io** **https://protocols.io/view/protocol-for-the-isolation-and-characterization-of-dj554q86**

**Created:** August 25, 2024

**Last Modified:** July 28, 2025

**Protocol Integer ID:** 106397

**Keywords:** Extracellular particles, Isolation and characterization, Brain

**Funders Acknowledgements:**
SENACYT (DR)
Grant ID: PFID-INF-2020-22
SENACYT (DR)
Grant ID: IDDS22-09
UP (DR)
Grant ID: CUFI-2023-CS-P-001
SENACYT (ADA)
Grant ID: APY-NI-2021-04
SENACYT (MD)
Grant ID: APY-NI-2023A-93

## Abstract

Extracellular particles (EPs) are a heterogeneous pool of secreted messengers in cell-cell communication. The isolation of EPs from supernatants, biofluids, and solid tissues allows further research into understanding the role of EPs in physiological and pathological scenarios, which aids in the development of EP-based therapies in biomedicine.

This article presents a straightforward, direct, and applicable method for isolating and characterizing EPs from three regions of the porcine brain: the cerebrum, cerebellum, and brainstem. The protocol is a method based on three steps: enzymatic treatment, differential centrifugation, and filtration/ultrafiltration to isolate the brain's EPs. Analysis by scanning electron microscopy (SEM) and nanoparticle tracking analysis (Zeta View) revealed the enrichment of the brain's EPs in the size range of 20 to 200 nm when isolated using this protocol. Additionally, CD63 and HSP70 expression was assessed by Western Blot without finding significant differences between the brain regions.

This simple adapted method will help the understanding of extracellular ecosystems in the CNS and could have interesting implications in brain diagnosis and therapy.

# Materials

**For EVs Isolation**

- Hibernate E-Ca Medium **Neuromics Catalog** #M36101-500ML
- Collagenase Type III from Clostridium histolyticum **MP Biomedicals Catalog** #215070401
- PBS [Phosphate Buffered Saline] **Research Products International Corp (RPI) Catalog** #P32200-1000.0
- PhosSTOP **Roche Catalog** #04906837001
- cOmplete™ ULTRA Tablets, Mini, EASYpack Protease Inhibitor Cocktail **Roche Catalog** #05892970001
- Amicon Ultra-0.5 Centrifugal Filter Unit - 10KDa cutoff **Merck Catalog** #UFC501024

**For Scanning Electron Microscopy:**

- Formvar Film 300 Mesh, Cu **Electron Microscopy Sciences Catalog** #FF300-Cu-50
- Uranyl acetate **Electron Microscopy Sciences Catalog** #22400
- Glutaraldehyde solution, Grade II, 25% in H2O **Merck MilliporeSigma (Sigma-Aldrich) Catalog** #G6257-100ML

**For Western blot**

- Ripa Buffer 2X Solution **Research Products International Corp (RPI) Catalog** #R26200-250.0
- Immobilon-P PVDF Membrane **Merck Catalog** #IPVH00010
- Pierce BCA Protein Assay Kit **Thermo Fisher Scientific Catalog** #23225 .
- Anti-CD63 antibody - Late Endosome Marker **Abcam Catalog** #ab134045
- Anti-Hsp70 antibody **Abcam Catalog** #ab181606
- Anti-NeuN Antibody, clone A60 **Merck MilliporeSigma (Sigma-Aldrich) Catalog** #MAB377
- Goat Anti-Rabbit IgG H&L (HRP) **Abcam Catalog** #ab6721

## Protocol materials

- Hibernate E-Ca Medium **Neuromics Catalog** #M36101-500ML
- Collagenase Type III from Clostridium histolyticum **MP Biomedicals Catalog** #215070401
- Glutaraldehyde solution, Grade II, 25% in H2O **Merck MilliporeSigma (Sigma-Aldrich) Catalog** #G6257-100ML
- 10% TGX Stain Free Fast Cast **Bio-Rad Laboratories Catalog** #1610183
- Uranyl acetate **Electron Microscopy Sciences Catalog** #22400
- PBS [Phosphate Buffered Saline] **Research Products International Corp (RPI) Catalog** #P32200-1000.0
- PhosSTOP **Roche Catalog** #04906837001
- Hibernate E-Ca Medium **Neuromics Catalog** #M36101-500ML
- Anti-NeuN Antibody, clone A60 **Merck MilliporeSigma (Sigma-Aldrich) Catalog** #MAB377
- Anti-Hsp70 antibody **Abcam Catalog** #ab181606
- Collagenase Type III from Clostridium histolyticum **MP Biomedicals Catalog** #215070401
- Pierce BCA Protein Assay Kit **Thermo Fisher Scientific Catalog** #23225
- Amicon Ultra-0.5 Centrifugal Filter Unit - 10KDa cutoff **Merck Catalog** #UFC501024
- Formvar Film 300 Mesh, Cu **Electron Microscopy Sciences Catalog** #FF300-Cu-50
- Uranyl acetate **Electron Microscopy Sciences Catalog** #22400
- Glutaraldehyde solution, Grade II, 25% in H2O **Merck MilliporeSigma (Sigma-Aldrich) Catalog** #G6257-100ML
- Ripa Buffer 2X Solution **Research Products International Corp (RPI) Catalog** #R26200-250.0
- Goat Anti-Rabbit IgG H&L (HRP) **Abcam Catalog** #ab6721
- cOmplete™ **Roche Catalog** #11873580001
- Immobilon-P PVDF Membrane **Merck Catalog** #IPVH00010
- Anti-CD63 antibody - Late Endosome Marker **Abcam Catalog** #ab134045
- PhosSTOP **Roche Catalog** #4906845001
- cOmplete™ **Roche Catalog** #11873580001
- Formvar Film 300 Mesh, Cu **Electron Microscopy Sciences Catalog** #FF300-Cu-50
- Laemmli buffer **Thermo Scientific Catalog** #1610747
- Immobilon-P PVDF Membrane **Merck Millipore (EMD Millipore) Catalog** #IPVH00010
- HRP Goat-Anti-Rabbit **Abcam Catalog** #ab6721
- RIPA **VWR International (Avantor) Catalog** #N653-100ML
- cOmplete™ **Roche Catalog** #11873580001
- PhosSTOP **Roche Catalog** #4906845001

- Pierce BCA **Thermo Scientific Catalog #**A65453
- Coomassie Stain **Research Products International Corp (RPI) Catalog #**B43000-25.0
- Red Ponceau **Abcam Catalog #**ab146313
- Anti-CD63 **Abcam Catalog #**ab134045
- Anti-HSP70 **Abcam Catalog #**ab181606
- Anti-NeuN **Merck MilliporeSigma (Sigma-Aldrich) Catalog #** Cat # MAB377
- PREMIUM Chemiluminiscent Substrate **LI-COR Catalog #**926-95010

# Isolating specific regions of the porcine brain

1 **Obtaining and storage**

2 The porcine brain tissues were extracted from 6 to 8-month-old porcine weighing approximately 118 kg immediately after being sacrificed at Marcello, S.A.

3 The brains were quicklyfrozen on dry ice and transferred to -80 °C for preservation, minimizing freeze-thaw degradation.

4 **Dissection**

5 After two days, the brains were thawed to remove blood vessels and were dissected to obtain three specific brain regions: the cerebrum, cerebellum, and brainstem.

6 Each dissected area was cut into small pieces, approximately 1 $cm^3$, for subsequent procedures.

7 Brain pieces were obtained for EP isolation.

8 Other brain pieces were pulverized in a dry mortar with a pestle to obtain homogenized tissue.

9 The development of experimental activities using these biological samples (porcine brains) adheres to the standards and criteria approved by exemption from the research protocol in the Animal Research and
Welfare Ethics Committee of the University of Panama.

10

CITATION

Reginensi D, Ortiz D, Pravia A, Burillo A, Morales F, Morgan C, Jimenez L, Dave KR, Perez-Pinzon MA, Gittens RA. (2020). Role of Region-Specific Brain Decellularized Extracellular Matrix on In Vitro Neuronal Maturation. Tissue Eng Part A.

LINK

https://doi.org/10.1089/ten.tea.2019.0277

CITATION

Reginensi D, Ortiz DA, Denis B, Castillo S, Burillo A, Khoury N, Xu J, Dam ML, Escobar AAH, Dave KR, Perez-Pinzon MA, Gittens RA (2025). Region-specific brain decellularized extracellular matrix promotes cell recovery in an in vitro model of stroke..

LINK

https://doi.org/10.1038/s41598-025-95656-w

## Isolation of EP from brain-region tissue

11 **Enzymatic treatment**

11.1 Brain pieces of porcine brain were randomly selected from three brain regions: the cerebrum, cerebellum, and brainstem. This pieces were sliced and treated with 75 U/ml of Collagenase Type III from Clostridium histolyticum **MP Biomedicals Catalog** #215070401 , in Hibernate E-Ca Medium **Neuromics Catalog** #M36101-500ML using a ratio of 800 µL of the solution for every 100 mg of brain tissue.

11.2 The mixture was incubated at 37 °C while shaking at 55 rpm for 00:20:00 . 20m

12 **Serial centrifugations**

12.1 Following this treatment, brain cells were removed by centrifuging the mixture at 300 x g for 00:05:00 at 4 °C . 5m

12.2 The supernatant was then transferred to a polycarbonate tube containing PhosSTOP **Roche Catalog** #4906845001 and cOmplete™ ULTRA Tablets, Mini, EASYpack Protease Inhibitor Cocktail **Roche Catalog** #05892970001 in phosphate-buffered saline.

12.3 This was centrifuged at 2000 x g for 00:10:00 at 4 °C to eliminate cellular debris. 10m

12.4 Next, to remove large debris and vesicles >200 nm, enriching for EPs within the 20–200 nm range, the supernatant underwent a second centrifugation at 10000 x g for 00:30:00 at 4 °C. 30m

13 **Filtration and ultrafiltration**

13.1 And then clarified through a 0.22 µm filter.

13.2 The clarified supernatant was placed in **10kDa** ultrafiltration tubes (Amicon; Cat # UFC901024; Millipore) and centrifuged at 5000 x g for 01:00:00 to concentrate the extracellular particles, with samples being mixed every 15 min during this process. 1h

14 Finally, the concentrated supernatant was washed three times by adding 15 mL of PBS and centrifuging at 5000 x g for 01:00:00 at 4 °C to obtain the EPs. 15m

15

CITATION

Vella LJ, Scicluna BJ, Cheng L, Bawden EG, Masters CL, Ang CS, Willamson N, McLean C, Barnham KJ, Hill AF. (2017). A rigorous method to enrich for exosomes from brain tissue. JOURNAL OF EXTRACELLULAR VESICLES.

LINK

https://doi.org/10.1080/20013078.2017.1348885

CITATION

Vergauwen, G., B. Dhondt, J. Van Deun, E. De Smedt, G. Berx, E. Timmerman, K. Gevaert, I. Miinalainen, V. Cocquyt, G. Braems, R. Van den Broecke, H. Denys, O. De Wever, and A. Hendrix. (2017). Confounding factors of ultrafiltration and protein analysis in extracellular vesicle research. Sci Rep.

LINK

https://doi.org/10.1038/s41598-017-02599-y

16

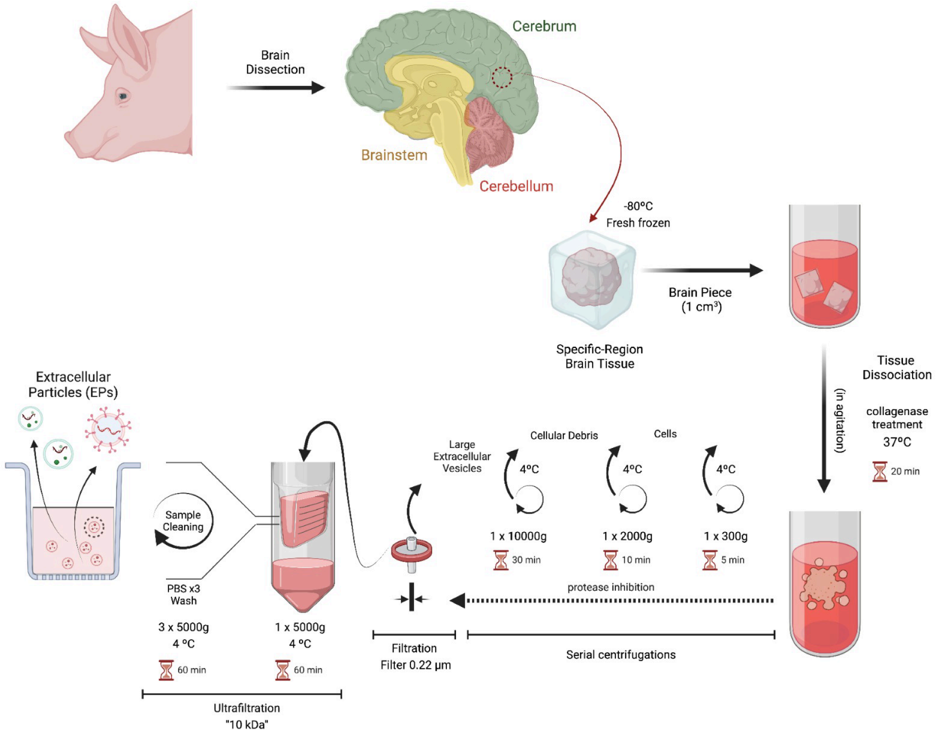


**Figure 1. Schematic of isolating extracellular particles (EPs).** EPs were isolated from porcine brain tissue through the following steps: (i) enzymatic treatment using collagenase type III for tissular dissociation, (ii) protease and phosphatase inhibition for ensuring protein integrity, (iii) differential centrifugation to remove tissue, cellular debris and large EPs, and (iv) filtration to enrich the sample in EPs smaller than 200 nm, (v) followed by ultrafiltration with a 10 kDa molecular weight cut-off to concentrate the sample and obtain extracellular particles from different brain regions (Created with Biorender.com, accessed Dec. 2024).

## Characterization of brain EPs by scanning electron microscopy (SEM)

1h 21m

17

Note

We mounted the brain EPs onto formvar square mesh grids

Formvar Film 300 Mesh, Cu **Electron Microscopy Sciences Catalog** #FF300-Cu-50

. Three different fixation protocols were evaluated: Keerthikumar's Protocol (Protocol A), Polanco's Protocol (Protocol B), and Lunavat's Protocol (Protocol C). Samples were charged onto the dark and shiny side of the grids, and the excess of liquid was paper blotted away between each step. Prior SEM analysis using the Quattro Environmental Scanning Electron Microscope (Thermo Fisher Scientific), all prepared grids were kept away from light sources.

18 **Protocol A (Keerthikumar's Protocol)**

18.1 10 µL of EPs sample was placed over TEM grids, for 00:05:00 . 5m

18.2 Excess residue was blotted away.

18.3 10 µL of 2% Uranyl acetate **Electron Microscopy Sciences Catalog** #22400 were pipetted twice over the grid for negative staining, for 00:01:00 . 1m

19

Expected result

Following Keerthikumar's protocol, a total of 436 EPs were acquired from 38 micrographs during the electron microscopy procedure. This total was distributed as follows: 90 EPs from the cerebrum, 149 EPs from the cerebellum, and 197 EPs from the brainstem.

19.1

CITATION

Keerthikumar, S., L. Gangoda, M. Liem, P. Fonseka, I. Atukorala, C. Ozcitti, A. Mechler, C.G. Adda, C.S. Ang, and S. Mathivanan. (2015). Proteogenomic analysis reveals exosomes are more oncogenic than ectosomes. Oncotarget.

LINK

https://doi.org/10.18632/oncotarget.3801

20 **Protocol B (Polanco's Protocol)**

20.1 Samples from each region were fixed using **2%** Glutaraldehyde solution, Grade II, 25% in $H_2O$ **Merck MilliporeSigma (Sigma-Aldrich) Catalog** #G6257-100ML for 00:30:00 . 30m

20.2 A 1:1 ratio of sample to fixation solution was prepared, and 10 µL of the fixed EP was added to the TEM grids, were it was allowed to absorb for five minutes.

20.3 Each grid was then washed twice with a droplet of sterile water.

20.4 After washing, 10 µL of 1.5% UA was applied for 1 min, ensuring it was protected from light.

21

Expected result

A total of 362 EPs distributed across 39 micrographs were acquired on the grids prepared according to Polanco's protocol. The distribution of EPs was as follows: 108 for the cerebrum, 128 for the cerebellum, and 126 for the brainstem.

21.1

CITATION

Polanco, J.C., B.J. Scicluna, A.F. Hill, and J. Götz. (2016). Extracellular Vesicles Isolated from the Brains of rTg4510 Mice Seed Tau Protein Aggregation in a Threshold-dependent Manner. J Biol Chem.

LINK

https://doi.org/10.1074/jbc.m115.709485

22 **Protocol C (Lunavat's Protocol)**

22.1 The grids were exposed to ultraviolet radiation for 00:15:00 on their dark-shiny side. 15m

22.2 EPs samples in a volume of 15 µL were incorporated into the grids for 00:10:00 . 10m

22.3 Samples were fixed by adding 15 µL of 2.5% glutaraldehyde for 00:10:00 . 10m

22.4 Fixed samples were stained with 15 µL of 2%UA, for 00:10:00 . 10m

23

**Expected result**

In total, 396 EPs were identified from 47 micrographs, distributed as follows: 137 EPs for the
cerebrum sample, 138 EVPs for the cerebellum sample, and 121 EPs for the brainstem.

23.1

**CITATION**

Lunavat, T.R., L. Cheng, D.K. Kim, J. Bhadury, S.C. Jang, C. Lässer, R.A. Sharples, M.D. López, J. Nilsson, Y.S. Gho, A.F. Hill, and J. Lötvall. (2015). Small RNA deep sequencing discriminates subsets of extracellular vesicles released by melanoma cells--Evidence of unique microRNA cargos. RNA Biol.

LINK

https://doi.org/10.1080/15476286.2015.1056975

24

**Note**

Images were taken in Quattro scanning electron microscope (SEM), in transmission mode, for all the samples prepared, operating at 30.00 kV.

The overall magnification was 50,000 x - 100,000 x with a current of 0,70nA and a working distance (WD) 9.3 mm to 10.9 mm .

To enhance visualization, Dark Field 4th ring and High Angle Annular Dark Field (HADF) modes were selected.

The electron microscopy was conducted at the Instituto de Investigaciones Cientificas y Servicios de Alta Tecnologia (INDICASAT-AIP), Panama.

25

Note

Ultrastructure measurements, included a total count of 334, 415 and 444 EPs from cerebrum, cerebellum, and brainstem, respectively.

The diameter of each EP was calculated as the median of a pair of diameters for each EP, for both round and elliptic EPs.

In this analysis, only samples that accomplish the selection criteria were included: defined borders, non-clustering and opacity due to the contrast given by the negative staining.

Quantification and measurement assessments were performed manually using ImageJ 1.54f. Samples were additionally classified by size in the following subcategories: non-vesicular extracellular particles (<50nm), small extracellular vesicles (50nm-200nm) and large extracellular vesicles (>200nm).

## Characterization of brain EPs by nanoparticle tracking analysis (NTA).

26 For NTA, the ZetaView (Particle Metrix, North Carolina, USA) instrumentation was validated using PS100 standard beads prior to the analysis of samples. Then, EPs sample were gently vortexed and centrifuged at 10000 rpm for 30 s.

27 Each sample was diluted in particle free water (MilliQ) for a total of 100 to 300 particles per field (Cerebrum EPs dilution factor: 50,000; Cerebellum EPs and Brainstem EPs dilution factor: 100,000).

28 For each measurement consider size distribution mode, 1 cycle and 11 positions with the following settings: (i) video resolution: High, (ii) laser wavelength: 488 nm, (iii) filter wavelength: scatter, (iv) camera sensitivity for all samples: 82.0, (v) shutter: 95, and (vi) cell temperature: 24°C.

29 After capture, analyze the videos by the in-build ZetaView Software 8.06.01 with specific analysis parameters: maximum particle area: 1000, minimum particle area: 6, and minimum particle brightness: 20

30

CITATION

Bachurski D, Schuldner M, Nguyen PH, Malz A, Reiners KS, Grenzi PC, Babatz F, Schauss AC, Hansen HP, Hallek M, Pogge von Strandmann E (2019). Extracellular vesicle measurements with nanoparticle tracking analysis – An accuracy and repeatability comparison between NanoSight NS300 and ZetaView. J Extracell Vesicles.

LINK

https://doi.org/10.1080/20013078.2019.1596016

## Characterization of EPs by Western Blot.

4h 29m

31 **Extracellular particle and brain homogenate tissue lysis**

31.1 For brain tissue, a small ~20mg piece was placed in PBS with inhibitors of phosphatase and protease, homogenized with a Wheaton tissue grinder and placed at -80°C for later use. Samples were lysed by radioimmunoprecipitation assay with RIPA lysis buffer RIPA **VWR International (Avantor) Catalog** #N653-100ML supplemented with protease cOmplete™ **Roche Catalog** #11873580001 and phosphatase PhosSTOP **Roche Catalog** #4906845001 inhibitors at 4 °C.

31.2 Samples were sonicated for 00:02:00 at Room temperature, and lysis was completed by incubation at 4 °C Overnight. 2m

31.3 Cellular debris and large EPs were then removed by centrifugation at 12000 rpm 4 °C for 00:05:00. 5m

32 **Sample preparation for loading**

32.1 Using the BCA assay kit Pierce BCA **Thermo Scientific Catalog** #A65453 , the overall protein concentration of the samples was determined. Prepare a diluted stock of the protein supernatant (e.g., 1:10 dilution) for analysis. Analyze the samples in duplicates. Protein concentrations may vary between samples (e.g., 2.04 – 4.12 µg/µl for native brain cerebrum); therefore, it is essential to perform the assay for each experiment. 20 µg of proteins were loaded onto each gel lane.

32.2 For the identification of tetraspanin proteins (e.g., CD63), the samples were prepared for loading using Laemmli buffer **Thermo Scientific Catalog** #1610747 without a reducing agent.

32.3 Laemmli buffer was diluted 1:1 with $dH_2O$ to prepare a 2x working stock. Prior to sample loading in the gel, protein samples were mixed with equal parts of the 2x Laemmli buffer to achieve a final 1x buffer concentration, and samples were heated in a dry bath incubator for 00:05:00 at 95 °C. 5m

32.4 For the identification of other proteins (e.g., HSP70, NeuN), the samples were additionally prepared with Laemmli buffer, including 355 mM 2-Mercaptoethanol (Cat # M3148-100ML; Sigma-Aldrich).

33 **Electrophoresis**

33.1 For electrophoresis samples were loaded onto 10% polyacrylamide gels 10% TGX Stain Free Fast Cast **Bio-Rad Laboratories Catalog #**1610183 . The parameters were set at 100V for 02:00:00 . 2h

34 **Transfer**

34.1 Proteins were transferred from the gel to PVDF membrane Immobilon-P PVDF Membrane **Merck Millipore (EMD Millipore) Catalog #**IPVH00010 with the following parameters: for two mini-gel, the transfer system (Transblot Turbo, Biorad) was set in one cassette at 25V, 1.0A for 01:00:00 . 1h

34.2 The gel was washed in $dH_2O$, and the total protein retained in the gel was visualized using Coomassie Stain **Research Products International Corp (RPI) Catalog #**B43000-25.0 . After transfer, the total protein was visualized using Red Ponceau **Abcam Catalog #**ab146313 stain.

35 **Antibody targetting**

35.1 The membrane was blocked with 5% fat-free milk (NFDM; Cat # 1706404; Biorad) in Tris-buffered
saline with Tween 20 (TBS-t) buffer for 01:00:00 at 100 rpm at room temperature.
Following this, the membrane was incubated with primary antibodies, which were diluted in 5% NFDM, for 16:00:00 at 100 rpm at 4 °C . After incubation, the membrane was washed three times with TBS-t.

Note

**Primary Antibodies used:**

**Extracellular vesicle particle markers (1:1000)**
Anti-CD63 **Abcam Catalog** #ab134045
Anti-HSP70 **Abcam Catalog** #ab181606

**Neuronal nuclei marker; non EV marker (1:1000)**
Anti-NeuN **Merck MilliporeSigma (Sigma-Aldrich) Catalog #** Cat # MAB377

35.2 Next, the membrane was incubated with a secondary antibody HRP Goat-Anti-Rabbit **Abcam Catalog** #ab6721 for 01:00:00 at 100 rpm at Room temperature . 1h

36 **Chemiluminescence for protein identification**

36.1 Afterward, the membrane was developed with a chemiluminescent substrate PREMIUM Chemiluminiscent Substrate **LI-COR Catalog** #926-95010 for 00:05:00 at Room temperature without shaking. 5m

36.2 Visualization was performed using a blot scanner (C-DiGit; Licor) in high resolution after a 00:12:00 exposure. 12m

## Statistical analysis

37 Data are presented as mean ± Standard Error of the Mean (SEM) of at least three independent experiments (unless indicated). Data was analyzed using one-way ANOVA with Tukey-Kramer post hoc
analysis ($p<0.05$). All analysis and graphing of results were performed using GraphPad Prism 10 software.

## Protocol references

Reginensi D, Ortiz D, Pravia A, Burillo A, Morales F, Morgan C, et al. Role of Region-Specific Brain Decellularized Extracellular Matrix on In Vitro Neuronal Maturation. Tissue Eng Part A. 2020;26(17-18):964-78. PubMed PMID: 32103711.

Reginensi D, Ortiz DA, Denis B, Castillo S, Burillo A, Khoury N, et al. Region-specific brain decellularized extracellular matrix promotes cell recovery in an in vitro model of stroke. Sci Rep. 2025;15(1):11921. PubMed PMID: 40195414.

Vella LJ, Scicluna BJ, Cheng L, Bawden EG, Masters CL, Ang CS, et al. A rigorous method to enrich for exosomes from brain tissue. J Extracell Vesicles. 2017;6(1):1348885. PubMed PMID: 28804598.

Vergauwen G, Dhondt B, Van Deun J, De Smedt E, Berx G, Timmerman E, et al. Confounding factors of ultrafiltration and protein analysis in extracellular vesicle research. Sci Rep. 2017;7(1):2704. PubMed PMID: 28577337.

Keerthikumar S, Gangoda L, Liem M, Fonseka P, Atukorala I, Ozcitti C, et al. Proteogenomic analysis reveals exosomes are more oncogenic than ectosomes. Oncotarget. 2015;6(17):15375-96. PubMed PMID: 25944692.

Polanco JC, Scicluna BJ, Hill AF, Götz J. Extracellular Vesicles Isolated from the Brains of rTg4510 Mice Seed Tau Protein Aggregation in a Threshold-dependent Manner. J Biol Chem. 2016;291(24):12445-66. PubMed PMID: 27030011.

Lunavat TR, Cheng L, Kim DK, Bhadury J, Jang SC, Lässer C, et al. Small RNA deep sequencing discriminates subsets of extracellular vesicles released by melanoma cells--Evidence of unique microRNA cargos. RNA Biol. 2015;12(8):810-23. PubMed PMID: 26176991.

Bachurski D, Schuldner M, Nguyen PH, Malz A, Reiners KS, Grenzi PC, et al. Extracellular vesicle measurements with nanoparticle tracking analysis - An accuracy and repeatability comparison between NanoSight NS300 and ZetaView. J Extracell Vesicles. 2019;8(1):1596016. PubMed PMID: 30988894.

## Citations


Step 10

Reginensi D, Ortiz D, Pravia A, Burillo A, Morales F, Morgan C, Jimenez L, Dave KR, Perez-Pinzon MA, Gittens RA.. Role of Region-Specific Brain Decellularized Extracellular Matrix on In Vitro Neuronal Maturation
**https://doi.org/10.1089/ten.tea.2019.0277**

Step 10

Reginensi D, Ortiz DA, Denis B, Castillo S, Burillo A, Khoury N, Xu J, Dam ML, Escobar AAH, Dave KR, Perez-Pinzon MA, Gittens RA. Region-specific brain decellularized extracellular matrix promotes cell recovery in an in vitro model of stroke.
**https://doi.org/10.1038/s41598-025-95656-w**

Step 15

Vella LJ, Scicluna BJ, Cheng L, Bawden EG, Masters CL, Ang CS, Willamson N, McLean C, Barnham KJ, Hill AF. . A rigorous method to enrich for exosomes from brain tissue
**https://doi.org/10.1080/20013078.2017.1348885**

Step 15

Vergauwen, G., B. Dhondt, J. Van Deun, E. De Smedt, G. Berx, E. Timmerman, K. Gevaert, I. Miinalainen, V. Cocquyt, G. Braems, R. Van den Broecke, H. Denys, O. De Wever, and A. Hendrix. . Confounding factors of ultrafiltration and protein analysis in extracellular vesicle research
**https://doi.org/10.1038/s41598-017-02599-y**

Step 19.1

Keerthikumar, S., L. Gangoda, M. Liem, P. Fonseka, I. Atukorala, C. Ozcitti, A. Mechler, C.G. Adda, C.S. Ang, and S. Mathivanan.. Proteogenomic analysis reveals exosomes are more oncogenic than ectosomes
**https://doi.org/10.18632/oncotarget.3801**

Step 21.1

Polanco, J.C., B.J. Scicluna, A.F. Hill, and J. Götz.. Extracellular Vesicles Isolated from the Brains of rTg4510 Mice Seed Tau Protein Aggregation in a Threshold-dependent Manner
**https://doi.org/10.1074/jbc.m115.709485**

Step 23.1

Lunavat, T.R., L. Cheng, D.K. Kim, J. Bhadury, S.C. Jang, C. Lässer, R.A. Sharples, M.D. López, J. Nilsson, Y.S. Gho, A.F. Hill, and J. Lötvall.. Small RNA deep sequencing discriminates subsets of extracellular vesicles released by melanoma cells--Evidence of unique microRNA cargos
**https://doi.org/10.1080/15476286.2015.1056975**

Step 30

Bachurski D, Schuldner M, Nguyen PH, Malz A, Reiners KS, Grenzi PC, Babatz F, Schauss AC, Hansen HP, Hallek M, Pogge von Strandmann E. Extracellular vesicle measurements with nanoparticle tracking analysis – An accuracy and repeatability comparison between NanoSight NS300 and ZetaView
**https://doi.org/10.1080/20013078.2019.1596016**

## Acknowledgements

DR was supported by Grants: PFID-INF-2020-22 and IDDS22-09 (SENACYT, Panama) and CUFI-2023-CS-P-001 (UP, Panamá) and funding of Nuevos Investigadores, SENACYT: APY-NI-2021-04 (ADA) and APY-NI-2023A-93 (MD). JM, MV-A and DR, we thank the support of the Panamanian National System of Researchers (SNI-SENACYT), Panama, Panama. Finally, this work was partially funded by Universidad Especializada de las Américas (UDELAS) under Academic-Administrative Agreement Nº 001-2024 and supported through the Centro de Investigaciones en Biotecnologia, Energias Verdes y Cambio Climático (BEVCC).
